# Secretome of hESC-Derived MSC-like Immune and Matrix Regulatory Cells Mitigate Pulmonary Fibrosis through Antioxidant and Anti-Inflammatory Effects

**DOI:** 10.3390/biomedicines11020463

**Published:** 2023-02-05

**Authors:** Wenfeng Hu, Jiali Yang, Jing Xue, Jia Ma, Shuang Wu, Jing Wang, Ranran Xu, Jun Wei, Yujiong Wang, Shuyan Wang, Xiaoming Liu

**Affiliations:** 1 Laboratory of Ministry of Education for Conservation and Utilization of Special Biological Resources of Western China, College of Life Science, Ningxia University, Yinchuan 750021, China; 2 Zephyrm Biotechnologies Co., Ltd., Beijing 102200, China; 3 Center Laboratory, Ningxia Clinical Research Institute, People’s Hospital of Ningxia Hui Autonomous Region, Yinchuan 750001, China; 4 Department of Anatomy and Cell Biology, Carver College of Medicine, University of Iowa, Iowa City, IA 52242, USA

**Keywords:** secretome, mesenchymal stem cells, human embryonic stem cells, pulmonary fibrosis, oxidative stress, inflammation, aging

## Abstract

Oxidative stress and inflammation are major drivers in the pathogenesis and progression of pulmonary fibrosis (PF). The mesenchymal stem cell (MSC) secretome has regenerative potential and immunomodulatory functions. Human embryonic stem cell (hESC)-derived MSC-like immune and matrix regulatory cells (IMRCs) are manufacturable with large-scale good manufacturing practice (GMP) preparation. In the present study, the antioxidative and anti-inflammatory properties and the therapeutic effect of the secretome of hESC-MSC-IMRC-derived conditioned culture medium (CM) (hESC-MSC-IMRC-CM) were investigated. Results revealed the capacities of hESC-MSC-IMRC-CM to reduce bleomycin (BLM)-induced reactive oxygen species (ROS), extracellular matrix (ECM) deposition, and epithelial–mesenchymal transition (EMT) in A549 cells. The administration of concentrated hESC-MSC-IMRC-CM significantly alleviated the pathogenesis of PF in lungs of BLM-injured mice, as accessed by pathohistological changes and the expression of ECM and EMT. A mechanistic study further demonstrated that the hESC-MSC-IMRC-CM was able to inhibit BLM-induced ROS and pro-inflammatory cytokines, accompanied by a reduced expression of Nox4, Nrf2, Ho-1, and components of the Tlr4/MyD88 signaling cascade. These results provide a proof of concept for the hESC-MSC-IMRC-derived secretome treatment of PF, in part mediated by their antioxidative and anti-inflammatory effects. This study thus reinforces the development of ready-to-use, cell-free hESC-MSC-IMRC secretome biomedicine for the treatment of PF in clinical settings.

## 1. Introduction

Idiopathic pulmonary fibrosis (IPF) is a chronic progressive lung disease hallmarked by fibroblast proliferation and accumulation of extracellular matrix (ECM), leading to irreversible distortion of the parenchymal architecture of the lung and respiratory failure [1]. To date, the median survival of IPF has been 3–5 years following diagnosis due to poor prognosis and limited therapeutic options to slow rather than reverse disease progression or restore pulmonary functions; therefore, innovative strategies and agents are unmet medical needs. Increasing evidence has allowed several factors to be postulated in the pathogenesis of pulmonary fibrosis, including oxidative stress [2], inflammation [3], and aging [4]. Among them, inflammation is regarded as the initiation of pulmonary fibrosis. Upon lung injury, inflammatory cells were activated and accumulated with the secretion of various inflammatory cytokines (such as TNF-α, IL-1β, and IL-17), which promote the proliferation and transformation of alveolar fibroblasts and the induction of ECM deposition [5]. Oxidative stress, which reflects an imbalance between the production of reactive oxygen species (ROS) and reactive nitrogen species (RNS), is one of the key factors implicated in the pathogenesis of IPF [6]. Accumulating evidence has demonstrated that inflammatory cells and epithelial cells in the lung tissue of IPF patients promote high levels of ROS production [7], NADPH oxidase (NOX) [8], and antioxidant depletion [9], which could exacerbate the pathogenesis of pulmonary fibrosis [6]. Therefore, targeting oxidative stress and inflammation may be key measures for developing novel therapeutic approaches to effectively prevent and/or treat chronic lung disease.

Mesenchymal stem cells (MSCs) are multipotent stem cells that emerge as a promising treatment approach in curing chronic and acute lung diseases by alleviating pulmonary fibrosis and improving pulmonary functions, due to their anti-inflammatory, antioxidative, regenerative, and immunomodulatory properties [10,11]. Human embryonic stem cell (hESC)-derived mesenchymal stem cell (MSC)-like immune and matrix regulatory cells (IMRCs) (hESC-MSC-IMRC) are a promising source of MSCs compared to other types of MSCs that are mainly derived from tissues, due to their genetic stability and reproducibility, as well as their potency in proliferation and differentiation [12,13], which exhibit great potential in treatments of several diseases, including IPF [14,15,16]. Importantly, hESC-MSC-IMRCs exhibit the advantages of unlimited availability with cell homogeneity, which are manufacturable for large-scale good manufacturing practice (GMP) preparation over conventional tissue-derived MSCs [14,16]. In vivo studies have further demonstrated that treatment with hESC-MSC-IMRCs is superior to that with primary umbilical cord mesenchymal stem cells (UCMSCs) or the FDA-approved pirfenidone in ameliorating the development of pulmonary fibrosis and inflammatory responses in the bleomycin (BLM)-induced IPF model [14,16]. In addition, clinical trials have shown that intravenous administration of IMRCs demonstrates significant effectiveness in the treatment of COVID-19 patients [16], and IMRC-derived extracellular vesicles (IMRC-EVs) showed a therapeutic effect on a bleomycin-induced pulmonary fibrosis mouse model [14]. However, it is well known that MSC-derived secretomes have up to 80% of the therapeutic effects of adult MSCs, which are released by MSCs, which are composed of extracellular vesicles (EVs) and soluble factors in the culture medium (CM) or released into the resident tissues [17,18]. The MSC-derived secretome is able to regulate various biologic processes via functions such as anti-inflammation, immunomodulation, anti-apoptosis, and antioxidation [18]. However, the application and potential of secretome in the medium of hESC-MSC-IMRCs (hESC-MSC-IMRC-CM) in the treatment of pulmonary fibrosis has not been reported, and the underlying therapeutic mechanism of hESC-MSC-IMRCs remains unclear.

In this report, we aimed to investigate the effects and underlying mechanisms of hESC-MSC-IMRC-CM on pulmonary fibrosis in a BLM-induced pulmonary fibrosis mouse model. We further analyzed the potential antioxidative and anti-inflammatory effects of hESC-MSC-IMRC-CM on pulmonary fibrosis in in vitro and in vivo models. Our results demonstrate that hESC-MSC-IMRC-CM could effectively alleviate pulmonary fibrosis through a mechanism of inhibiting oxidative stress and inflammation. 

## 2. Materials and Methods

### 2.1. Secretome Containing hESC-MSC-IMRC Culture Medium (hESC-MSC-IMRC-CM) 

Both hESC-MSC-IMRCs and hESC-MSC-IMRC-CM were kindly prepared and provided by Zephyrm Biotechnologies (Beijing, China). The passage 5 of hESC-MSC-IMRCs were seeded at 2 × 10^4^ cells/cm^2^ in a T225 cell culture flask with 35 mL medium and refreshed with serum-free culture medium every other day [14,16]. The hESC-MSCs were passaged at about 80–90% confluence and harvested at passage 7 for further study. To prepare the hESC-MSC-IMRC-CM, the passage 5 of hESC-MSC-IMRCs (designated as MSCs in this report) were propagated to 50% confluence and rinsed with pre-warmed PBS prior to being cultured with refreshed medium for an additional 48 h. The medium was then collected and centrifuged at 3000 rpm/min for 10 min to remove cell debris. The resultant supernatant was concentrated 5-fold using Merck Amicon™ Ultra-15 Centrifugal Filter Units devices (Millipore, Burlington, MA, USA) at 4 °C, 4000× *g* for 60 min, to cutoff 3-kDa molecular weight. The concentrated supernatant was collected as hESC-MSC-IMRC-CM (designated as CM in this report), which was aliquoted and frozen at −80 °C till it was used. 

### 2.2. Animal Welfare Statement and Procedures 

The Laboratory Animal Committee of Ningxia University, in accordance with guidelines of the National Institutes of Health Guide for the Care and Use of Laboratory Animals, approved all mice studies (NXULS20180123–3). Six- to eight-week-old C57BL/6 male mice were purchased from Beijing Vital River Lab Animal Technology Co., Ltd. (Beijing, China) and housed in a room with a 12/12 h light/dark cycle with food and water *ad libitum*. To assess the effect of hESC-MSC-IMRC-CM in alleviating the pulmonary fibrosis processes, the animal experimental design and treatment was schemed in Figure S1. In the study of the antifibrotic effect of bone marrow stromal cells (BMSCs)-CM in rats, 1 mL of CM was injected through the tail vein for treatment [19], and the dose of CM volume (200 µL) required by mice in this study was thus converted by body weight compared to rat. Wu and collages have shown that single intravenous hESC-MSC-IMRCs mitigate BLM-induced pulmonary fibrosis in a dose-dependent manner (1 × 10^6^ cells, 3 × 10^6^ cells and 5 × 10^6^ cells) [14,16]. To avoid the death caused by pulmonary embolism during the tail vein administration with high-dose cell preparation, a medium dose of hESC-MSC-IMRCs (3 × 10^6^ cells) was thus selected as the number of cells that were administrated by 2 separate doses (1.5 × 10^6^ cells in 100 μL saline per dose) with a 1 h interval. The dose of BLM used for the induction of IPF in mice has been previously described with slight modification [20]. Briefly, the mice were challenged with bleomycin (BLM, 1.5 mg/kg body weight) (MedChemExpress, Monmouth Junction, NJ, USA) or saline by intratracheal instillation via laryngotracheal route at day 0 (d0) and d7. For an early intervention for mice at the early stage (Figure S1A) of pulmonary fibrosis with the hESC-MSC-IMRCs or hESC-MSC-IMRC-CM at the early stage of pulmonary fibrosis development, 3 × 10^6^ cells of hESC-MSC-IMRCs or 200 μL of concentrated hESC-MSC-IMRC-CM were administrated via tail vein injection at d3, d7, and d14 after the first dose of BLM challenge. For the intervention at the late stage of pulmonary fibrosis (Figure S1B), hESC-MSC-IMRCs or hESC-MSC-IMRC-CM was administrated at d7, d14, and d21. The mice in the control groups without intervention were injected with the same volume of saline to match the hESC-MSC-IMRCs or hESC-MSC-IMRC-CM group. The lung tissues were collected at d28 after the first dose of BLM challenge.

### 2.3. Cell Culture and BLM-Induced A549 Alveolar Epithelial Cells Injury 

Human adenocarcinoma alveolar epithelial cell line A549 (ATCC CCL-185) was purchased from American Type Culture Collection (ATCC) (Manassas, VA, USA) and cultured in DMEM supplemented with 10% Fetal Bovine Serum (FBS) and 1% penicillin/streptomycin at 37 °C in a humidified atmosphere of 5% CO_2_. A549 cells (5 × 10^5^ cells/well) were seeded into a 6-well plate and cultured for 12 h, and the medium containing various concentrations (0, 1.0, 2.5, 5.0, 10.0 and 20.0 μg/mL) of BLM or/and hESC-MSC-IMRC-CM was replaced, and the cells were cultured for an additional 48 h. Then, the cells were harvested for analysis.

### 2.4. Lung Index 

The body weight (BW) and the lung weight (LW) of mice were recorded at the termination of experiment (28th day of treatment), and the ratio of LW/BW was served as a lung index that was calculated by the equation: Lung index = LW/BW*100.

### 2.5. Lung Histopathological and Immunohistochemical Analysis

Mouse lung tissues were fixed in 4% paraformaldehyde solution (PFA) for 72 h, before they were processed for embedding in an Auto-embedding machine (JB-P5, Wuhan Junjie Electronics Co., Ltd., Wuhan, China). Hematoxylin and eosin (H&E) staining and Masson staining were performed on 5 μm thick paraffin sections. H&E staining and Masson staining were performed using H&E dye solution set (Servicebio, G1003, Wuhan servicebio technology Co., Ltd., Wuhan, China) and Masson dye solution set (Servicebio, G1006, Wuhan servicebio technology Co., Ltd., Wuhan, China), respectively. For immunofluorescent (IF) staining, the paraffin sections were deparaffinized in xylene and rehydrated in a series of gradient ethanol and boiled in citrate buffer (pH 6.0) at 95 °C for 15 min, and then cooled down to room temperature (RT) for antigen retrieval. The sections were treated with 0.3% Triton X-100/0.3% of H_2_O_2_ in PBS for 30 min. After rinsing with PBS twice, the sections were blocked with 5% donkey serum in PBS for 1 h at RT. Then, the sections were stained with 1:200 rabbit monoclonal IgG Heme oxygenase-1 (HO-1) (Proteintech, 27282-1-AP), rabbit monoclonal IgG nuclear factor erythroid 2–related factor 2 (NRF2) (Proteintech, 16396-1-AP), rabbit monoclonal IgG smooth muscle actin (SMA) (Proteintech, 14395-1-AP), rabbit monoclonal IgG NADPH Oxidase 4 (NOX4) (BOSTER, BA2813), rabbit monoclonal IgG Activating Transcription Factor 6 (ATF6) (Proteintech, 24169-1-AP), rabbit monoclonal IgG N-cadherin (Proteintech, 22018-1-AP), and mouse monoclonal IgG2b TNF-α (Proteintech, 60291-1-IG) overnight at 4 °C in 5% donkey serum in PBS. After washing with PBS three times, the sections were incubated with 1:500 Alexa Flour fluorescence 565-conjugated secondary antibodies in 5% donkey serum in PBS for 1.5 h at RT. Then, the nuclei were counterstained with DAPI (H-1200, Vector Laboratories, Burlingame, CA) for visualizing and imaging using Olympus IX73 and processed on Olympus CellSens Entry 2.3 (Olympus, Tokyo, Japan).

### 2.6. Hydroxyproline Content in Mice Lung Tissues

In order to detect the collagen content in the lung, the right lobes of the lung were used for a hydroxyproline assay (Nanjing Jiancheng Bioengineering institute, China) according to the manufacturer’s protocol. The hydroxyproline content was presented in micrograms (µg) per milligram of lung tissues.

### 2.7. Immunoblotting Analysis 

Whole cell extracts from lung tissues and A549 cells were used for RIPA lysis buffer (PS0012, Leagene Biotech, Beijing, China) and Whole Cell Lysis Assay (KGP2100, KeyGEN BioTECH, Nanjing, China) according to the manufacturer’s protocol, respectively. Thirty micrograms of total protein was resolved by 8–12.5% sodium dodecyl sulfate-(SDS-) poly-acrylamide gel (SDS-PAGE). The separated proteins were then transferred onto a PVDF nitrocellulose membrane. After blocking with 5% non-fat milk or 3% BSA in PBS for 1 h at RT, the membrane was incubated overnight at 4 °C with primary antibodies: rabbit monoclonal IgG HO-1 (27282-1-AP, Proteintech, Rosemont, IL, USA), rabbit monoclonal IgG NRF2 (Proteintech, 16396-1-AP), rabbit monoclonal IgG SMA (14395-1-AP, Proteintech, Rosemont, IL, USA), rabbit monoclonal IgG NOX4 (BA2813, Boster, Wuhan, China), mouse monoclonal IgG NOX2 (BA2811, Boster, Wuhan, China), mouse monoclonal IgG anti-α-tubulin (T5168, SIGMA, St. Louis, MO, USA), rabbit monoclonal IgG ATF6 (24169-1-AP, Proteintech, Rosemont, IL, USA), rabbit monoclonal IgG N-cadherin (22018-1-AP, Proteintech, Rosemont, IL, USA), rabbit monoclonal IgG NOX3 (20065-1-AP, Proteintech, Rosemont, IL, USA), rabbit monoclonal IgG Toll-like receptor 4 (TLR4) (BS20594R, Bioss, Woburn, MA, USA), and rabbit monoclonal IgG Myeloid differentiation primary response 88 (MyD88) (NB100-56698, Novusbio, Centennial, CO, USA). Then, the membrane was washed with 1 × PBS containing 0.2% Tween-20 three times and incubated for 1.5 h with appropriate (HRP)-conjugated secondary antibodies for 1 h at RT. The membrane was developed to enhance chemiluminescence (ECL) reagent (Advansta, Menlo Park, CA, USA). The levels of protein expression were semi-quantified by optical densitometry using ImageJ Software version 1. The relative expression of the protein of interest was normalized by the GAPDH internal loading control and presented as densitometric arbitrary units (A.U.). 

### 2.8. Measurement of Oxidative Stress and ROS Staining 

Measures of 40–50 mg of right lobes of mouse lung were taken, ground into homogenate, and centrifuged, and the soluble fraction was collected for subsequent determination. Both lipid peroxidation malondialdehyde (MDA) and the activity of glutathione peroxidase (GSH-PX) were accessed using assay kits per the manufacturer’s instructions from Beyotime Biotechnology (Shanghai, China). For ROS Staining, cells (1 × 10^5^/well) were seeded in a 12-well plate and cultured for 12 h and then treated with various concentrations of BLM (0, 1.0, 2.5, 5.0, 10.0 and 20.0 μg/mL) for an additional 48 h. Subsequently, 200 μL of 5 μmol/L CellROX™ Orange solution (C10443, Thermo Fisher) was added to each well, and the plate was incubated with light proof at RT for 30 min. Images were captured and photographed with Olympus IX73 (Olympus, Japan). 

### 2.9. ELISA for TNF-α, IL-1β, IL-6

The lung tissues were homogenized with RIPA lysis buffer (PS0012, Leagene, Beijing, China). The cell lysates were harvested and measured with BCA Protein Assay Kit (Keygen Biotech, Nanjing, China) to detect the protein concentration. Then, the soluble protein solutions were added into the wells, which were analyzed for the levels of TNF-α, IL-1β, and IL-6 with mouse TNF-α, IL-1β, and IL-6 ELISA kits (Cat no. m1002095, m1063132, m1002293, Shanghai MLBIO Biotechnology Co. Ltd., Shanghai, China). 

### 2.10. Statistical Analysis

The data in this study are expressed as mean ± standard deviation (SD). All analyses were performed using PRISM 8.0.1 (GraphPad Software, La Jolla, CA, USA) and performed by One-way or Two-way ANOVA Tukey’s multiple comparisons test. *p* < 0.05 was considered significant.

## 3. Results

### 3.1. An Elevated Oxidative Stress and Tlr4/MyD88 Signaling Activity in Lungs of BLM-Induced PF Mice 

**Figure 1:**
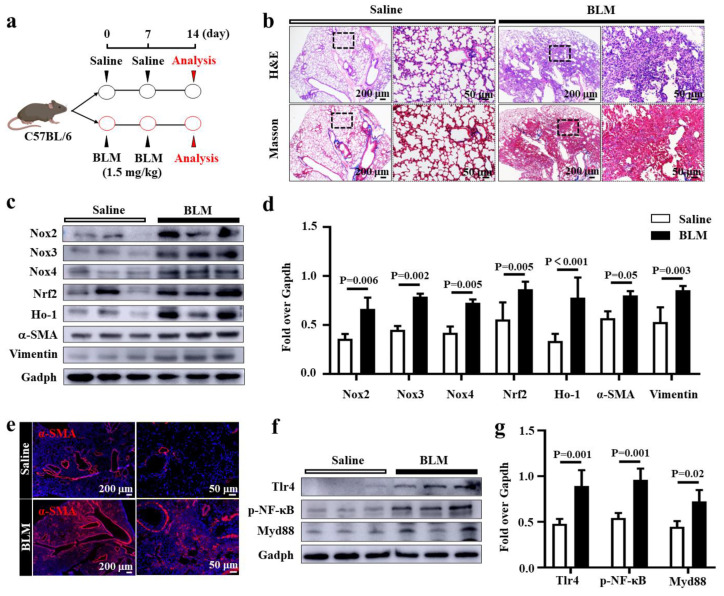
Elevated Nox-mediated oxidative stress and Tlr4/MyD88-mediated inflammation in lungs of mice with BLM-induced pulmonary fibrosis (PF). (**a**) Schematic diagram for the creation of the BLM-induced PF mouse model. C57BL/6 mice that were 6–8 weeks old were injured by intratracheal instillation of 1.5 mg/kg BLM at day 0 (d0) and d7, and the lung tissues were harvested at d14 post-injury for evaluation (N = 8). (**b**) Representative histochemical images of H&E and Masson staining demonstrated alveolar structural damage (H&E staining) with ECM deposition (Masson staining). (**c**) Representative immunoblotting blots demonstrated an increased expression of proteins of Nox family numbers (Nox2, Nox3, and Nox4), oxidization stress markers (Nrf2 and Ho-1), and EMT markers (α-SMA and Vimentin) in the lungs of BLM-induced PF mice compared with the saline group. (**d**) Semi-quantitation of relative levels of proteins of interest as determined by the relative density over Gapdh in (**c**). (**e**) IF staining validated the increased abundance of α-SMA in the lungs of mice at d14 post-BLM challenge compared with saline controls. (**f**) Representative blots of immunoblotting assay demonstrated the enhanced activation of Tlr4/MyD88 signaling in the lung of BLM-induced PF mice model compared with the saline group. (**g**) Semi-quantitation of the relative levels of proteins of interest Tlr4, MyD88, and p-NF-κB as evaluated by the relative density over Gapdh in (**f**). Data in (**d**,**g**) represent mean ± SD from the indicated number of mice (N = 3), as analyzed by GraphPad prism one-way ANOVA Tukey’s multiple comparisons test.

In order to investigate the pathogenic roles of oxidative stress and inflammation in the development of PF, the expression of key components of signaling cascades of oxidative stress and inflammation, including antioxidative factors such as Nrf2, Ho-1, and Nox family numbers and the key molecules of TLR/MyD88 signaling cascade, were examined in the lungs of BLM-induced PF mice by immunoblotting and immunofluorescence (IF) assays (Figure 1). The mouse model of PF was generated by the instillation of BLM in the lung (Figure 1a) and evaluated by H&E and Masson staining (Figure 1b). Pathohistological evaluation of lung tissues at day 14 post BLM challenge showed alveolar structural damages with markedly thickened lung interstitium in BLM-induced lung tissue compared to control mice treated with saline (Figure 1b). Masson staining further demonstrated abundant extracellular matrix (ECM) deposition in BLM-injured lungs (Figure 1b). Moreover, molecular analysis revealed an increased expression of Nox2, Nox3, Nox4, Nrf2, and Ho-1, coupled with abundant EMT markers alpha-smooth muscle actin (α-SMA) and vimentin in lung tissues of mice challenged with BLM compared to the saline group (Figure 1c,d). Furthermore, the increased α-SMA was further corroborated in lung tissue of mice treated with BLM, as evaluated by IF staining (Figure 1e). These results demonstrate that aberrant oxidative stress and the family of Noxs are involved in the pathogenesis of PF. As expected, increased Tlr4, MyD88, and Phospho-NF-κB p65 were also observed in BLM-induced mouse PF lungs, as ascertained by immunoblotting assay (Figure 1f,g). These results indicate the success in creating BLM-injured mouse PF model for further studies of interventions. These data also demonstrate that both signaling pathways of oxidative stress Tlr4/MyD88-mediated inflammation were activated in the lungs of mice during the pathogenesis of PF, which led us to hypothesize that targeting oxidative stress and inflammation may be a key measure for developing novel therapeutic approaches to effectively prevent and/or treat PF.

### 3.2. BLM Induces Oxidative Stress and NOX4-Mediated ROS Production in Lung Epithelial Cells

**Figure 2:**
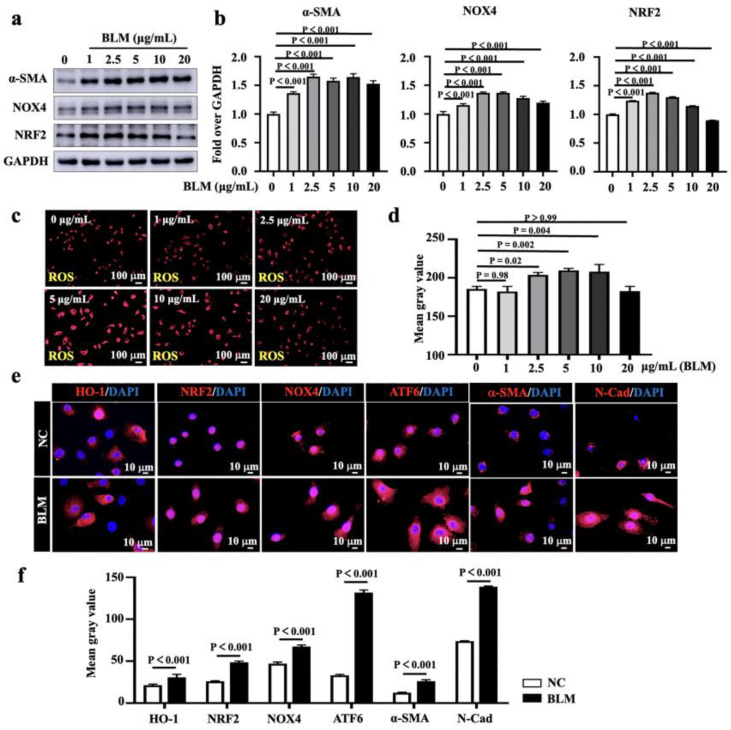
BLM induces NOX4-mediated oxidative stress in A549 lung epithelial cells. (**a**) Representative images of immunoblotting blots revealed an increased expression of α-SMA, NOX4 and NRF2 in A549 cells exposed to indicated concentrations of BLM (1.0, 2.5, 5.0, 10.0, and 20.0 μg/mL) for 48 h. (**b**) Semi-quantitation of the relative levels of α-SMA, NOX4, and NRF2 proteins as evaluated by the relative density over GAPDH in (**a**). (**c**) Representative images of A549 cells treated with indicated concentrations of BLM for 48 h and stained with CellROX^®^ Orange reagent. The BLM-induced A549 injury increased ROS production, especially at the concentration of 5 μg/mL, compared to untreated cells (NC). (**d**) Mean gray value of ROS production in (**c**). (**e**) Representative images of IF exhibited more abundant HO-1, NRF2, NOX4, ATF6, α-SMA, and N-Cad proteins in A549 cells exposed to 5 μg/mL of BLM for 48 h as compared with the NC control. (**f**) Representative images of the fluorescent intensity of the relevant proteins in (**e**). Data in (**b**,**d**,**f**) represent mean ± SD from three independent experiments (N = 3), as analyzed by GraphPad prism one-way ANOVA Tukey’s multiple comparisons test. Scale bars in (**c**), 100 µm; (**e**), 10 µm.

Oxidative stress is one of the key features of the pathogenesis of PF [2]. NOX proteins are major sources of ROS production, and NOX4 is an inducible source of intracellular ROS of diseased heart [21] and lungs [22,23]. NOX4 has been demonstrated to have an implication in the development and progression of PF [24] and is a potential target for the treatment of acute lung injury [25]. To evaluate whether oxidative stress and NOX4 are involved in A549 lung epithelial cells in response to exposure to BLM, the alterations of oxidative stress markers and NOX4 were examined. Following the A549 cells exposed to BLM at different concentrations ranging from 0 to 20 μg/mL (0, 1.0, 2.5, 5.0, 10.0, and 20.0 μg/mL) for 48 h, the exposure to BLM led to significantly increased abundances of α-SMA, NOX4, and NRF2 expression compared to untreated (NC) cells (Figure 2a,b). Furthermore, the BLM-induced A549 injury increased ROS production, especially in the concentration of 5 μg/mL, compared to untreated control cells (Figure 2c,d). Immunofluorescence analysis of the expression of oxidative stress-related markers and EMT signatures revealed that BLM could significantly induce the expression of HO-1, NRF2, NOX4, ATF6, α-SMA, and N-Cad (Figure 2e,f). These data are concurrent with the notion that oxidative stress is the main driving force behind the development of pulmonary disease [2,6].

### 3.3. hESC-MSC-IMRC-CM Treatment Suppresses the BLM-Induced Oxidative Stress in Lung Epithelial Cells

**Figure 3:**
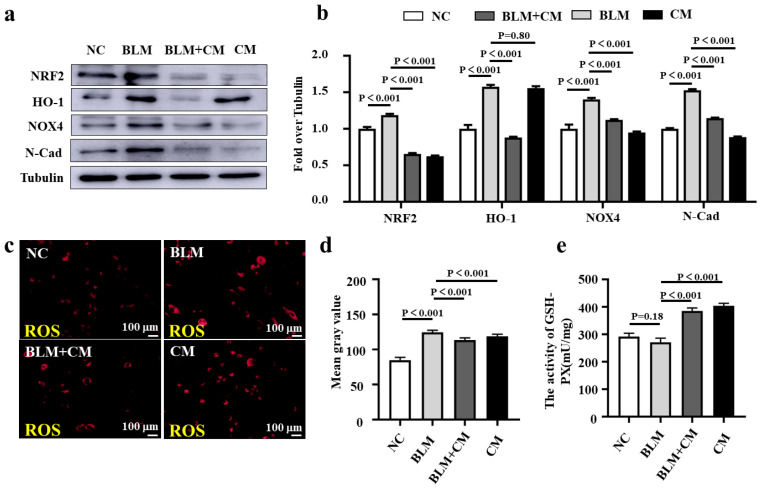
hESC-MSC-IMRC-CM reduces the BLM-induced oxidative stress and EMT in A549 lung epithelial cells. (**a**) Representative immunoblots showed that hESC-MSC-IMRC-CM could reduce BLM-induced A549 cells oxidative injury by decreasing the expression of NRF2, HO-1, NOX4, and EMT markers’ N-Cad. (**b**) The relative levels of NRF2, HO-1, NOX4, and N-Cad proteins as evaluated by a densitometric analysis in (**a**). (**c**) Representative images of A549 cells treated with indicated conditions for 48 h and stained with CellROX^®^ Orange reagent. hESC-MSC-IMRC-CM significantly reduced the ROS production compared to the BLM group. (**d**) Mean gray value of ROS production in (**c**). (**e**) hESC-MSC-IMRC-CM increased glutathione peroxidase (GSH-PX) production in A549 cells compared to untreated controls. Data in (**b**,**d**,**e**) represent mean ± SD of three independent experiments (N = 3), as analyzed by GraphPad prism Two-way ANOVA Tukey’s multiple comparisons test. Scale bars in (**c**), 100 μm.

To test the antioxidant properties of hESC-MSC-IMRC-CM, we exposed them to BLM-induced oxidative stress in A549 lung epithelial cells. We found that the NOX4 expression was significantly inhibited, accompanied by decreased abundances of NRF2 and HO-1 in BLM-treated A549 cells that were cultured in the presence of hESC-MSC-IMRC-CM, compared with cells with BLM treatment alone (Figure 3a,b). In addition, the expression of EMT marker α-SMA was also decreased in cells cultured with hESC-MSC-IMRC-CM as measured by immunoblotting assay (Figure 3a,b). Subsequential analysis of the fluorescent integrated intensity of ROS production revealed that the presence of hESC-MSC-IMRC-CM significantly reduced ROS production in the BLM-treated cells compared to the cells treated with BLM alone (Figure 3c,d). A consistent finding was that the addition of hESC-MSC-IMRC-CM apparently enhanced the activity of glutathione peroxidase (GSH-PX) (Figure 3e), one of the most important antioxidant enzymes of ROS [2], compared to control BLM-treated cells. These results suggest that hESC-MSC-IMRC-CM has an antioxidant property able to suppress BLM-induced oxidative stress in lung epithelial cells.

### 3.4. hESC-MSC-IMRCs and hESC-MSC-IMRC-CM Mitigate BLM-Induced PF in Mice 

**Figure 4:**
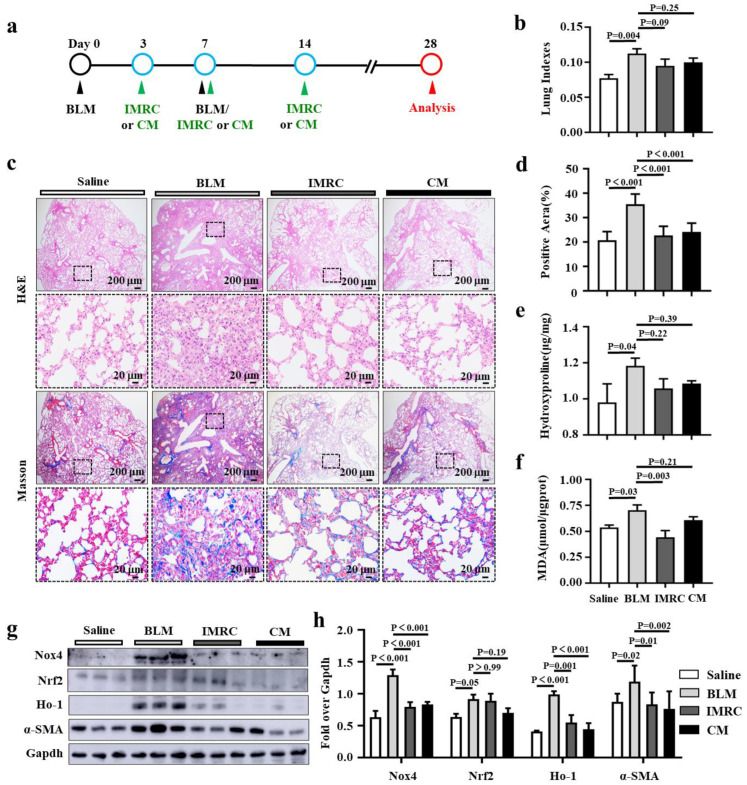
hESC-MSC-IMRCs and hESC-MSC-IMRC-CM inhibit the development of pulmonary fibrosis in mice. (**a**) Schematic illustration shows the experimental workflow for the treatment of the BLM-induced IPF mouse model at an early stage of PF development. C57BL/6 mice were challenged with BLM via the laryngotracheal route at day (**d**) 0 and d7. A 200 μL sample of saline containing 3 × 10^6^ cells of hESC-MSC-IMRCs and 200 μL of hESC-MSC-IMRC-CM were delivered via the tail vein injection at d3, d7, and d14 after the first dose of BLM challenge. The lung tissues were harvested at d28 for analysis. (**b**) The effect of hESC-MSC-IMRC-CM on lung index (ratio of lung/body weights). (**c**) Representative histochemical images of H&E and Masson staining demonstrate that both hESC-MSC-IMRC-CM and hESC-MSC-IMRCs can ameliorate the pathogenesis of pulmonary fibrosis (H&E staining) with ECM deposition (Masson staining) compared to animals challenged with BLM alone. (**d**) Histological analysis of the fibrotic area. (**e**,**f**) The levels of hydroxyproline (Hyp) and malondialdehyde (MDA) contents. (**g**) Representative immunoblots show a reduction in indicated molecules of oxidative stress-signaling pathways and α-SMA in BLM-induced fibrotic lung administrated with hESC-MSC-IMRC-CM or hESC-MSC-IMRCs compared to the lungs of BLM-challenged mice alone. (**h**) The relative levels of Nox4, Nrf2, Ho-1 and α-SMA proteins as evaluated by a densitometric analysis in (**g**). Data in (**b**,**d**–**f**,**h**) represent mean ± SD of three independent experiments (N = 3), as analyzed by GraphPad prism two-way ANOVA Tukey’s multiple comparisons test. Scale bars in (**c**), 200 µm in the first and third panels, 20 µm in the second and fourth rows.

**Figure 5:**
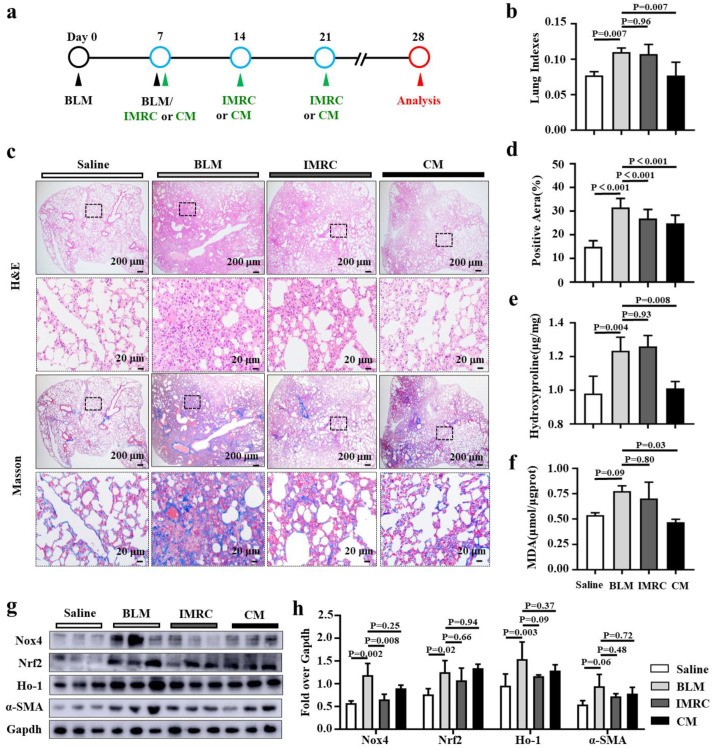
hESC-MSC-IMRC-CM alleviates the progression of pulmonary fibrosis in mice. (**a**) Schematic illustration shows the experimental workflow for treatments of late-stage of PF disease in the BLM-induced PF mouse model. C57BL/6 mice were challenged with BLM via the laryngotracheal route at day (**d**) 0 and d7. A 200 μL sample of saline containing 3 × 10^6^ cells of hESC-MSC-IMRCs or 200 μL hESC-MSC-IMRC-CM was administrated via tail vein injection at d7, d14, and d21 after the first dose of BLM challenge. The lung tissues were harvested at d28 for analysis. (**b**) The ratio of lung weight/body weight (LW/BW). (**c**) Representative histochemical images of H&E and Masson staining demonstrate that both hESC-MSC-IMRC-CM and hESC-MSC-IMRCs can ameliorate the progression of pulmonary fibrosis (H&E staining) with ECM deposition (Masson staining) compared to the BLM-group. (**d**) Histological analysis of the fibrotic area. (**e**,**f**) The level of hydroxyproline (Hyp) and malondialdehyde (MDA) contents. (**g**) Representative immunoblots show a decreased expression of indicated components of oxidative stress signaling cascade and α-SMA in BLM-induced lung treated with hESC-MSC-IMRC-CM or hESC-MSC-IMRCs compared to the untreated BLM group. (**h**) The relative levels of Nox4, Nrf2, Ho-1, and α-SMA proteins as evaluated by a densitometric analysis in (**g**). Data in (**b**,**d**–**f**,**h**) represent mean ± SD of three independent experiments (N = 3), as analyzed by GraphPad prism two-way ANOVA Tukey’s multiple comparisons test. Scale bars in (**c**), 200 µm in the first and third rows, 20 µm in the second and fourth rows.

A compelling body of studies has shown that MSCs could reduce lung fibrosis and restore lung function in clinical and pre-clinical settings [26,27]. Secretome derived from MSCs has been demonstrated to be attributable to the immunoregulatory and antioxidative potential of MSCs, which is believed to exert their paracrine and autocrine functions. In view of the fact that hESC-MSC-IMRCs and their EVs can significantly inhibit the development of pulmonary fibrosis and inflammatory responses in BLM-induced IPF models [14,16], we therefore aimed to evaluate the effectiveness of hESC-MSC-IMRC-CM in preventing/alleviating the development of pulmonary fibrosis in a BLM-induced murine model at both the early stage (Figure 4) and the late stage of disease development (Figure 5). We firstly investigated the effect of treatment that began at an early stage of PF pathogenesis (3 days post BLM challenge) in a mouse model of pulmonary fibrosis by the delivery of hESC-MSC-IMRC-CM or hESC-MSC-IMRCs via tail vein injection at designated time points (day 3, day 7, day 14) (Figure 4a). As shown in Figure 4b, a significant increase in lung indexes was observed in BLM-induced mice compared with those instilled with saline. The administration of both hESC-MSC-IMRC-CM and hESC-MSC-IMRCs slightly but not significantly decreased lung indexes compared to the BLM challenge alone (Figure 4b). In the histological evaluation by H&E staining, the lung tissues from BLM-induced mice showed focal fibrosis in comparison with the saline group (Figure 4c). The treatment of hESC-MSC-IMRC-CM or hESC-MSC-IMRCs significantly reduced fibrotic lesion in the lungs of mice challenged with BLM in comparison with the untreated BLM-injured animals, suggesting that both hESC-MSC-IMRC-CM and hESC-MSC-IMRCs have the potential to mitigate pulmonary fibrosis in mice challenged with BLM (Figure 4c). Masson staining, which was used to evaluate the collagen fibers, further revealed that the area of lung tissues with blue-stained ECM deposition was extensively observed in the BLM group compared to the saline group (Figure 4c,d). It is worth noting that the blue-stained ECM deposited areas were apparently reduced in the lungs of BLM-injured mice with hESC-MSC-IMRC-CM or hESC-MSC-IMRCs treatment relative to mice with BLM challenge alone (Figure 4c,d). In line with the histopathological findings, the content of hydroxyproline (Hyp) was also significantly reduced in lung tissues of mice treated with either hESC-MSC-IMRC-CM or hESC-MSC-IMRCs compared to those with BLM challenge alone (Figure 4e). Of interest, the Hyp level was not statistically different between mice treated with hESC-MSC-IMRC-CM and hESC-MSC-IMRCs (Figure 4e). To assess the contribution of antioxidative properties of hESC-MSC-IMRC-CM and hESC-MSC-IMRC in PF treatment, the level of malondialdehyde (MDA), a final product of lipid peroxidation and a biomarker of oxidative stress [6] was examined (Figure 4f). The MDA level was significantly increased in the lungs of BLM-challenged mice as compared to the saline group. Notably, the administration of hESC-MSC-IMRCs significantly decreased the MDA content compared to the BLM group, while the hESC-MSC-IMRC-CM treatment only moderately reduced MDA content, but there was no statistical difference compared to BLM challenge alone. Aligned with the decrease in MAD, molecular analysis revealed a significant inhibition of the expression of Nox4, Nrf2, Ho-1, and α-SMA in the lungs of BLM-challenged mice with hESC-MSC-IMRC-CM or hESC-MSC-IMRCs treatment compared to untreated BLM-injured mice (Figure 4g,h).

Using a similar approach, we next examined the effectiveness of hESC-MSCLs-CM treatment that started at the late stage in BLM-induced pulmonary fibrosis mice (7 days post BLM challenge) (Figure 5a). Of great interest, the administration of both hESC-MSC-IMRC-CM significantly decreased the lung indexes compared to other groups, although the hESC-MSC-IMRCs had no effect on the reduction in lung index (Figure 5b). The pathohistological evaluation demonstrated the effectiveness of hESC-MSC-IMRCs and hESC-MSC-IMRC-CM administrated at 7 days post BLM challenge, when the PF pathological changes were established. BLM-injured mice treated with hESC-MSC-IMRCs or hESC-MSC-IMRC-CM exhibited a lower severity of the pathology of pulmonary fibrosis compared with untreated BLM animals (Figure 5c), which was further corroborated by Masson staining and quantitative fibrotic score analysis (Figure 5c,d). The administration of hESC-MSC-IMRC-CM significantly reduced the BLM-induced collagen deposition (Figure 5c,d) and contents of Hyp (Figure 5e) and MDA (Figure 5f) in lungs of PF mice compared with untreated BLM-injured mice and mice treated with hESC-MSC-IMRCs. These data suggest that hESC-MSC-IMRC-CM could alleviate BLM-induced pulmonary fibrosis at both the early stage and the late stage of pathogenesis, which can at least in part be attributed to its antioxidant property.

### 3.5. hESC-MSC-IMRCs or hESC-MSC-IMRC-CM Alleviates Lung Inflammation in PF Mice 

**Figure 6:**
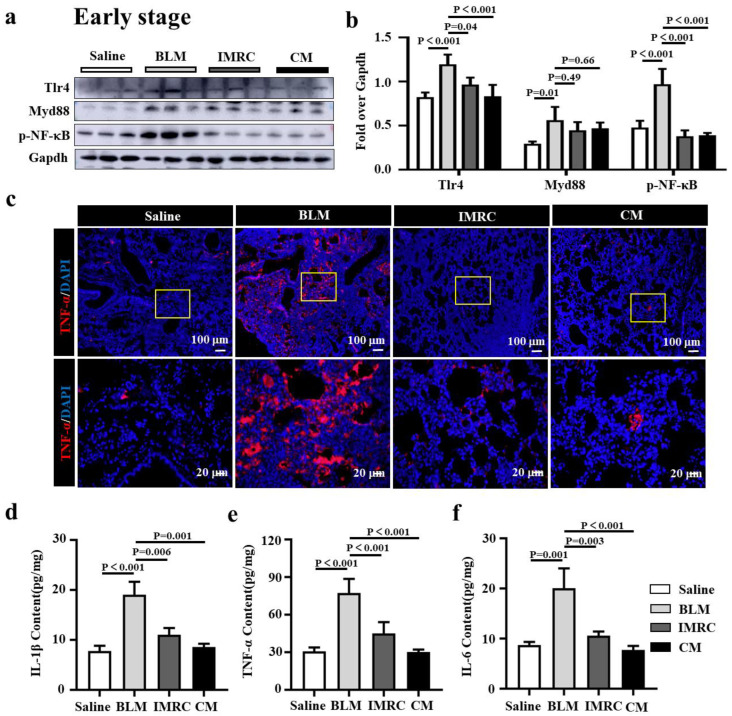
hESC-MSC-IMRCs and hESC-MSC-IMRC-CM ameliorate inflammation in mice in the development stage of PF. (**a**) Representative immunoblots showed a reduction of molecules in the Tlr4/MyD88 signaling cascade in BLM-induced lung treated with hESC-MSC-IMRC-CM or hESC-MSC-IMRCs at d3, d7, and d14 after the first dose of BLM challenge compared to the untreated BLM group. (**b**) The relative levels of Tlr4, MyD88, and p-NF-κB proteins as evaluated by a densitometric analysis in (**a**). (**c**) IF staining showed an increased abundance of TNF-α in BLM-induced pulmonary fibrosis compared to the saline group, but a decreased expression of TNF-α in the lungs of mice injected with hESC-MSC-IMRC-CM or hESC-MSC-IMRCs compared to the untreated BLM-injured mice. (**d**–**f**) the concentrations of TNF-α (**d**), IL-1β (**e**), and IL-6 (**f**) proteins in lung homogenates in BLM-injured mice as determined by ELISA. Data in (**b**,**d**–**f**) represent mean ± SD of three independent experiments (N = 3), as analyzed by GraphPad prism two-way ANOVA Tukey’s multiple comparisons test. Scale bars in (**c**), 100 µm in the top panels, 20 µm in the bottom panels.

**Figure 7:**
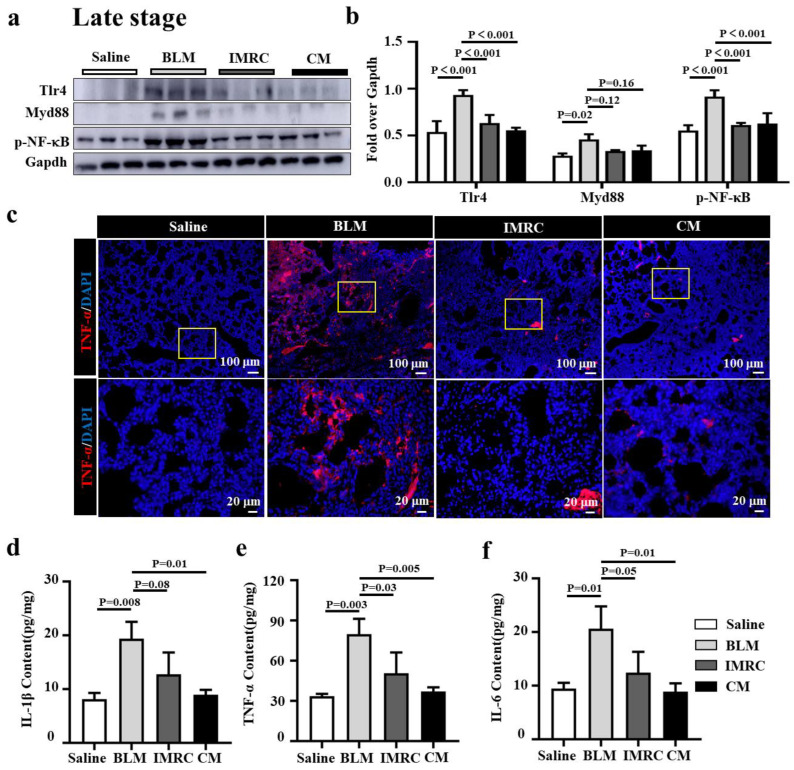
hESC-MSC-IMRCs and hESC-MSC-IMRC-CM attenuate inflammation in mice in the progressive stage of PF. (**a**) Representative immunoblots showed a reduction of molecules in the Tlr4/MyD88 signaling cascade in BLM-injured lungs administrated with hESC-MSC-IMRC-CM or hESC-MSC-IMRCs at d7, d14, and d21 after the first dose of BLM challenge, compared to BLM injury alone. (**b**) The relative levels of Tlr4, MyD88, and p-NF-κB proteins as evaluated by a densitometric analysis in (**a**). (**c**) IF staining showed an increased abundance of TNF-α in BLM-induced pulmonary fibrosis compared to the saline group, but a decreased expression of TNF-α in the lungs of mice injected with hESC-MSC-IMRC-CM or hESC-MSC-IMRCs compared to the untreated BLM groups. (**d**–**f**) The concentrations of TNF-α (**d**), IL-1β (**e**), and IL-6 (**f**) proteins in lung homogenates in BLM-injured mice as determined by ELISA. Data in (**b**,**d**–**f**) represent the mean ± SD of three independent experiments (N = 3), as analyzed by GraphPad prism two-way ANOVA Tukey’s multiple comparisons test. Scale bars in (**c**), 100 µm in the top panels, 20 µm in the bottom panels.

Inflammation is an initial driver of PF, and toll-like receptor 4 (TLR4)/MyD88-mediated inflammation and inflammatory cytokines have shown a profibrotic effect in the development of pulmonary fibrosis [5]. Next, we sought to investigate whether the anti-inflammatory function of MSCs contributed to the alleviation of PF in mice treated with hESC-MSC-IMRCs or hESC-MSC-IMRC-CM. As expected, remarkably elevated inflammation with the activation of TLR signaling and cytokine productions was observed, as ascertained the abundance of proteins Tlr4, Myd88, and p-NF-κB in BLM-injured lungs by immunoblotting assay and IF staining, and proinflammatory cytokines IL-1β, TNF-α and IL-6 in lung homogenates by ELISA in BLM-injured mice, compared to the saline treatment (Figure 6). The administration of hESC-MSC-IMRCs or hESC-MSC-IMRC-CM at d3, d7, and d14 post the first dose of BLM challenge led significant suppression of the BLM-induced expression of Tlr4, and p-NF-κB, TNF-α proteins (Figure 6a–c) and the production of IL-1β, TNF-α, and IL-6 (Figure 6d–f) in lungs of PF mice at an early stage of PF development, compared with that of mice challenged with BLM alone. It is worth noting that the administration of hESC-MSC-IMRC-CM and hESC-MSC-IMRCs injection could slightly but not significantly decrease Myd88 compared to BLM treatment challenge alone (Figure 6a,b). Importantly, the administration of hESC-MSC-IMRCs or hESC-MSC-IMRC-CM demonstrated the inhibitory effects on the amelioration of BLM-induced inflammatory responses in a late stage of PF progression (treatment was conducted at d7, d14, and d21 after the first dose of BLM challenge) by suppressing the expression of Tlr4, Myd88, and p-NF-κB and TNF-α protein, as assessed by immunoblotting assay (Figure 7a,b) and immunofluorescence staining of TNF-α (Figure 7c), and the secretion of IL-1β, TNF-α, and IL-6 in lungs of PF mice determined by ELISA (Figure 7d–f). These results clearly demonstrate that the anti-inflammatory properties of hESC-MSC-IMRC-CM and hESC-MSC-IMRCs are critical for their therapeutic effects in the treatment of PF in mice at both stages of early development and late progression of the disease, which is in part through the mechanism of regulating the Tlr4/MyD88 signaling pathway.

## 4. Discussion

It has been well established that the use of mesenchymal stem cells (MSCs) and their secreted factors (also named MSCs secretome) is an excellent therapeutic strategy for chronic and acute pulmonary diseases, based on a variety of animal models of lung injury [16,19]. It has been reported that up to 80% of the therapeutic effects of adult tissue-derived MSCs are executed by the MSC-derived secretome, which shows great promise in the treatments of chronic diseases, including IPF. 

The potential of hESC-MSC-IMRCs in the treatment of PF has been recently demonstrated [14,16]. In the present report, the effect and underlying mechanism of the secretome of hESC-MSC-IMRCs in culture medium (hESC-MSC-IMRC-CM) in the treatment of pulmonary fibrosis have been evaluated in a BLM-injured PF murine model. Our study demonstrated an aberrant activation of oxidative stress and inflammation signaling in the lungs of BLM-induced PF mice. Both hESC-MSC-IMRC-CM and its producing cells hESC-MSC-IMRCs were capable of scavenging BLM-induced ROS and inhibiting BLM-induced inflammatory responses, resulting in a reduction in ECM deposition and EMT in vitro and in vivo. The systemic administration of concentrated hESC-MSC-IMRC-CM or GMP-grade preparation of hESC-MSC-IMRCs was able to mitigate the development and progression of PF in the lungs of BLM-injured mice. Mechanistically, the underlying mechanism of actions of hESC-MSC-IMRC-CM was through its roles in regulating signaling pathways involved in Nox-mediated oxidation and Tlr4/MyD88-mediated inflammations. Our results further evidenced that hESC-MSC-IMRCs-derived secretome has a therapeutic potential in the treatment of pulmonary fibrosis.

Both the elaboration of inflammatory cytokines and oxidative stress (such as ROS) are significant drivers in the development of pulmonary fibrosis. Our results found that both hESC-MSC-IMRCs and hESC-MSC-IMRC-CM have an ability to alleviate BLM-induced pulmonary fibrosis by reducing oxidative stress and inflammation. Importantly, we also found that the therapeutic effect of hESC-MSC-IMRC-CM was superior to that of its producing parent cells, hESC-MSC-IMRCs, particularly when the hESC-MSC-IMRC-CM was administrated at a late stage of PF pathogenesis in mice. The hESC-MSC-IMRC-CM can significantly inhibit BLM-induced pulmonary fibrosis in mice by inhibiting the production of ROS and proinflammatory cytokines by regulating Nox4/Nrf2 and Tlr4/MyD88 signaling pathway, respectively.

Accumulating evidence has demonstrated that oxidative stress is an important mediator of lung injury, which can induce direct toxicity, including ROS generation, antioxidant depletion, and NADPH oxidase (NOX) expression, subsequentially leading to inflammatory responses and fibrosis in lungs [7,8,9,28,29]. The key enzymatic sources that produce ROS in lung include nitric oxide synthase (NOS), mitochondrial respiratory enzymes, xanthine oxidoreductase, and nicotinamide adenine dinucleotide phosphate (NADPH) oxidase (NOX). Indeed, our data suggest that oxidative stress is required for the development of PF in mice by inducing ROS production, accompanied by a remarkable increase in Ho-1 and Nrf2 expression in BLM-induced models of pulmonary fibrosis in vivo and in vitro. 

In this report, we firstly evaluated the effect of BLM-induced pulmonary fibrosis in vivo and in vitro by employing BLM-treated A549 alveolar epithelial cells as a model with a BLM in the dosage range of 0–20 µg/mL for 48 h. We found a dose-dependent effect of BLM (from 0 to 10 µg/mL) on ROS production, with a striking increase in α-SMA and NRF2 in A549. This finding was further confirmed in BLM-induced pulmonary fibrosis of a mouse model in vivo. Notably, hESC-MSC-IMRC-CM alone was also able to induce intracellular ROS production in A549 cells, although the CM could reduce the BLM-induced ROS production (Figure 3c,d). We speculated that hESC-MSC-IMRC-CM (secretome) might be able to induce ROS production in the absence of other exogenous oxidative stress such as BLM and promote A549 cell proliferation in vitro. Since A549 cells are an adenocarcinoma cell line with tumorigenicity, ROS production was required for tumor cell proliferation [30]. Generally, both NRF2 and heme oxygenase 1 (HO-1) were elevated upon oxidative stress [31]. Interestingly, although NRF2 was downregulated following the hESC-MSC-IMRC-CM treatment, its dependent gene *GPX* was unexpectedly upregulated in BLM-treated A549 cells, as seen in IPF lungs in which the expression of several antioxidant enzymes, including GPX, was increased as a part of the adaptive mechanism for excessive oxidative stress [32]. 

NADPH oxidases (NOX) are particularly important enzymes for ROS generation. Seven different NOXs homologues, namely are Nox1, Nox2, Nox3, Nox4, Nox5, Duox1, and Duox2, have been identified in mammals [33]. Recent studies have shown that NOX2 and NOX4 play a critical role in the pathogenesis of PF [8]. In the present study, we found that the expression of many isoforms of NOXs, especially Nox2, Nox3, and Nox4, were increased in BLM-challenged mouse lung. Among them, Nox4 has been demonstrated to play an important role in driving pulmonary fibrosis [34,35]. It has been well documented that an aberrant expression of NOX4 was observed in pulmonary fibroblasts of IPF patients and experimental IPF animal models [34,35]. Therefore, NOX4 has been considered a potential therapeutic target for the treatment of pulmonary fibrosis. Indeed, our results show that hESC-MSC-IMRC-CM can significantly alleviate BLM-induced A549 injury by decreasing the BLM-induced NOX4 and elevating NRF2 antioxidant activity in vitro. 

It has been reported that MSC-CM has the capability of preventing pulmonary fibrosis by an anti-inflammatory mechanism [36]. Increasing evidence has shown that IL-1β, IL-6, and TNF-α contribute to the pathogenesis of pulmonary fibrosis [5]. Indeed, one main etiology of pulmonary fibrosis is acute and/or chronic inflammation via the release of lung proinflammatory cytokines and chemokines (such as TNF-α, IL-1β, IL-6, and IL-8) along with NF-κB [37]. In this study, the administration of hESC-MSC-IMRCs or hESC-MSC-IMRC-CM exhibited an ability to suppress TNF-α, IL-1β, and IL-6 release in lung tissues of BLM-challenged mice, suggesting that hESC-MSC-IMRCs or hESC-MSC-IMRC-CM could mitigate pulmonary fibrosis by relieving inflammatory responses. This notion was in agreement with the finding that the anti-inflammatory effects of hESC-MSC-IMRCs can be used to treat severely ill COVID-19 patients with various lung diseases [16]. Intravenous transfusion of hESC-MSC-IMRCs showed a significant reduction in pro-inflammatory cytokine production in two patients with acute lung injury (ALI) [16]. Our previous study also demonstrated that the injection of human placental MSCs of fetal origins (hfPMSCs) could significantly alleviate BLM-induced pulmonary fibrosis with the decrease in Hyp and pro-fibrotic cytokines in mice by inhibiting the TLR/MyD88 signaling pathway [38]. Consistent with the therapeutic effect of hfPMSCs, both hESC-MSC-IMRCs and hESC-MSC-IMRC-CM displayed abilities to suppress inflammation by inhibiting the expression of TLR4, MyD88, and p-NF-κB and the production of proinflammatory cytokines TNF-α, IL-1β and IL-6.

It is worth noting that although the administration of either hESC-MSC-IMRCs or hESC-MSC-IMRC-CM showed a potential to mitigate BLM-induced pulmonary fibrosis in mice, the efficacy of MSCs therapies is mainly attributed to the MSC secretome in pulmonary diseases [18]. Consistently, our results presented in this report show that administration of hESC-MSC-IMRC-CM showed a better therapeutic effect in the treatment of PF over the direct administration of its producing cells hESC-MSC-IMRCs, as assessed by the reduction in the expression of fibrotic signature proteins, hydroxyproline content, and MDA content, as well as pathological fibrosis score, lung/body weight ratio. These data reinforce the notion that the secretome of MSCs is a major part of the therapeutic effect in the treatment of chronic diseases such as pulmonary fibrosis [18]. 

## 5. Conclusions

Collectively, in this report, we show that elevated oxidative stress and increased inflammation were observed in the lungs of BLM-injured PF mice. Intravenous delivery of hESC-MSC-IMRCs and hESC-MSC-IMRC-CM displayed therapeutic potencies of alleviating pulmonary fibrosis in PF mice, as evaluated by the decrease in fibrotic lesions, ECM deposition, EMT, and hydroxyproline content in lungs. Moreover, the administration of hESC-MSC-IMRCs had better effectiveness than hESC-MSC-IMRC-CM in mice with an early stage of PF pathogenesis at 3 days post BLM challenge. Intriguingly, hESC-MSC-IMRC-CM exhibited a superior therapeutic effect to hESC-MSC-IMRCs in mice with a late stage of PF at 7 days post BLM challenge. The hESC-MSC-IMRC-CM-mediated amelioration of pulmonary fibrosis was through a mechanism by suppressing the BLM-induced oxidative stress and inflammation via respective Nox4/Nrf2 and Toll/MyD88 signaling pathways. Thus, this report provides proof-of-concept evidence that hESC-MSC-IMRC-CM may be a promising therapeutic strategy for the treatment of pulmonary fibrosis.

## Data Availability

All data generated or analyzed during this study are included in this published article.

## Supplementary Materials

The following supporting information can be downloaded at https://www.mdpi.com/article/10.3390/biomedicines11020463/s1: Figure S1: Schema of experimental workflow for treatments in the BLM-induced IPF mouse model.
