# Supplementary material for: Secretome of hESC-Derived MSC-like Immune and Matrix Regulatory Cells Mitigate Pulmonary Fibrosis through Antioxidant and Anti-Inflammatory Effects
Source: Biomedicines. 2023 Feb 5;11(2):463. doi: 10.3390/biomedicines11020463 (PMC9953085; doi:10.3390/biomedicines11020463)
Supplement: Supplementary file 1 [file biomedicines-11-00463-s001.zip › biomedicines-2156969-supplementary.pdf]

Supplemental Files

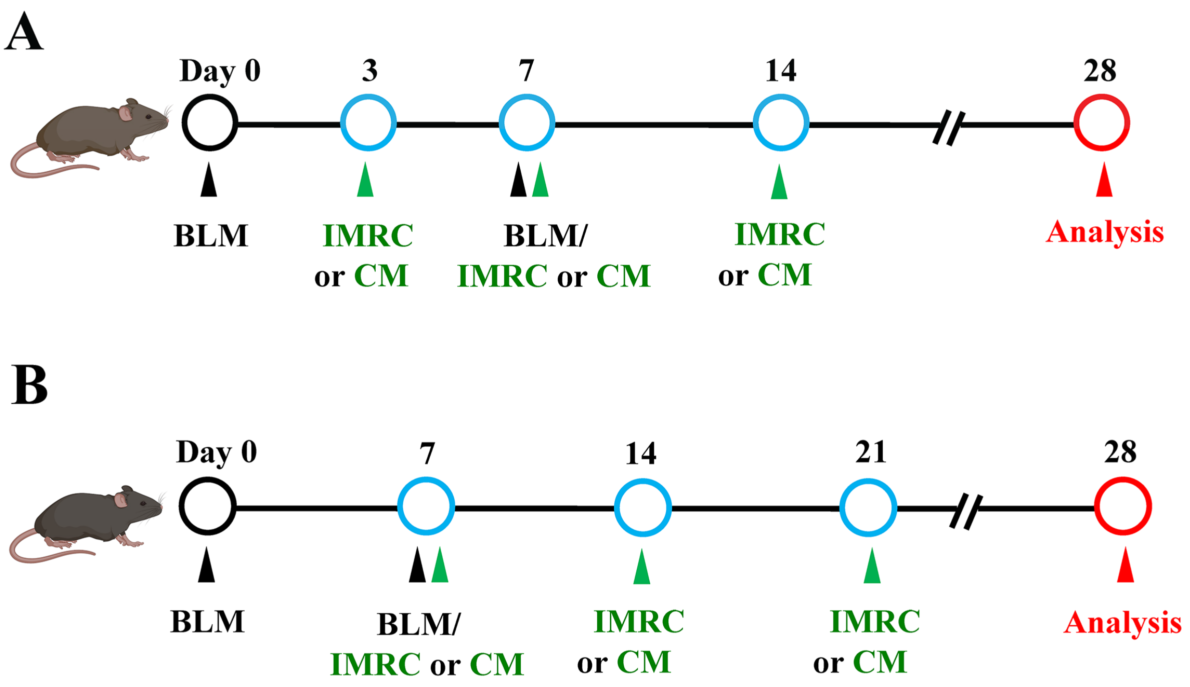

Suppl. Figure S1. Schema of experimental workflow for treatments in the BLM-induced IPF mouse model. (a) Schematic illustration shown the experimental workflow for the treatment of the BLM-induced IPF mouse model at an early stage of PF development. C57BL/6 mice were challenged with BLM via laryngotracheal route at day (d) 0 and d7. 200 µL saline containing 3 × 106 cells of hESC-MSC-IMRCs or 200 µL hESC-MSC-IMRC-CM were delivered via the tail vein injection at d3, d7 and d14 post the first dose of BLM challenge. The lung tissues were harvested at d28 for analysis. (b) Schematic illustration shown the experimental workflow for treatments of late-stage of PF disease in the BLM-induced IPF mouse model. C57BL/6 mice were challenged with BLM via laryngotracheal route at day (d) 0 and d7. 200 µL saline containing 3 × 106 cells of hESC-MSC-IMRCs or 200 µL hESC-MSC-IMRC-CM were administrated via tail vein injection at d7, d14 and d21 post the first dose of BLM challenge. The lung tissues were harvested at d28 for analysis. BLM=Bleomycin, CM=conditional medium, hESC-MSC-IMRC=human embryonic stem cells (hESCs)-derived mesenchymal stem cell (MSC)-likes immune and matrix regulatory cells (IMRCs) (hESC-MSC-IMRC), IPF=Idiopathic pulmonary fibrosis, PF=pulmonary fibrosis.
